# Comparison of assessments of fitness to drive for people with dementia

**DOI:** 10.1080/09602011.2014.903197

**Published:** 2014-05-06

**Authors:** Kristina Vella, Nadina B. Lincoln

**Affiliations:** ^a^ Institute of Work, Health and Organisations, University of Nottingham, Nottingham, UK; ^b^ Division of Rehabilitation and Ageing, University of Nottingham, Queens Medical Centre, Nottingham, UK

**Keywords:** Cognition, Driving, Dementia, Assessment

## Abstract

Cognitive tests are used to inform recommendations about the fitness to drive of people with dementia. The Rookwood Driving Battery (RDB) and Dementia Drivers' Screening Assessment (DDSA) are neuropsychological batteries designed to assist in this process. The aim was to assess the concordance between the classifications (pass/fail) of the RDB and DDSA in individuals with dementia, and to compare any discordant classifications against on-road driving ability. Participants were identified by community mental health teams and psychiatrists. Twenty four participants were recruited. The mean age was 74.1 (*SD* 8.9) years and 18 (75%) were men. Each participant was assessed on the RDB and DDSA in an order determined by random allocation. Those with discrepant results also had an on-road assessment. The agreement between the tests was 54% using a cut-off of > 6 on the RDB, and 75% using the cut-off to > 10 on the RDB. Three participants with discrepant results agreed to be assessed on the road and all were found to be safe to drive. The findings suggested that there was poor concurrent validity between the RDB and DDSA. This raises questions about the choice of assessments in making clinical recommendations about fitness to drive in people with dementia.

## INTRODUCTION

In clinical practice, healthcare professionals are asked to advise on whether people with dementia are safe to continue driving. In the UK, people with dementia are required to notify the Driver and Vehicle and Licensing Agency (DVLA) of their diagnosis and are then subject to review of their licence on an annual basis. The DVLA asks the medical person responsible for their care, usually a general practitioner or psychiatrist, for further details of their cognitive decline. Other countries have similar procedures. Occupational therapists and psychologists are also asked for information about the patient's cognitive abilities in order to inform the decision. Wilson and Pinner (2013) pointed out that the challenge is assessing the level of risk of a driver with dementia and determining the level of risk that is unacceptable. They also pointed out that this assessment relies on psychiatrists' clinical opinions, requiring yes/no answers to statements such as “Does your patient lack insight and/or judgement to a degree that would make driving dangerous?” Given the subjectivity in this process, attempts have been made to develop more standardised procedures. This may include the use of cognitive tests, but there is only limited evidence to support the use of cognitive testing (Iverson et al., 2010).

Research has shown an association between performance on cognitive tests and the ability to drive on the road. Reger et al. (2004) conducted a systematic review of studies examining the relation between neuropsychological function and driving ability in people with dementia. They identified 27 studies, 12 of which used on-road driving to assess driving ability. Neuropsychological tests were grouped according to the cognitive domains assessed. Effect sizes were significant but small for the relation between on-road driving and all neuropsychological tests in patients with dementia. When tests were classified according to the cognitive domain assessed, effect sizes were greatest for measures of visuospatial skills. Molnar, Patel, Marshall, Man-Son-Hing, and Wilson (2006) conducted a similar review but, instead of aggregating tests into cognitive domains, they examined each test separately. They identified 16 studies that examined the relation between cognitive tests and driving ability, but only six used on-road driving as the measure of driving ability. There were marked inconsistencies between studies, with tests showing positive associations with driving in some but not in others. They also identified the problem that very few studies provided cut-off scores for tests, which could be used to make clinical decisions with individual patients.

Cognitive tests are used for two purposes in relation to driving. One is to screen people attending memory clinics to identify those who need on-road assessment, as in some countries not every driver with dementia will be assessed on the road. People who have mild cognitive impairment will be allowed to continue to drive; those with very severe cognitive impairment will be deemed unsafe and advised to stop driving (Molnar et al., 2006; Wilson & Pinner, 2013). The purpose of cognitive screening is to identify those with borderline cognitive abilities, and to refer them for specialist on-road assessment. In addition, cognitive tests are used at specialist driving assessment centres as part of the overall evaluation and are used in conjunction with on-road assessment to make recommendations about safety to drive.

Two batteries of clinical tests have been validated as predictors of safety to drive for people with dementia and are used in clinical practice. They provide cut-off scores for making recommendations about safety to drive. The Rookwood Driving Battery (RDB; McKenna, 2009) was developed in the context of a specialist driving assessment centre. People with a range of neurological conditions, including dementia, were assessed on a battery of cognitive tests and performance on these tests was compared with the overall decision by the centre on participants' fitness to drive (McKenna, Jefferies, Dobson, & Frude, 2004; McKenna & Bell, 2007). Cut-off scores were developed to predict those people who were found to be unsafe to drive. For the sample as whole, a cut-off of > 10 was recommended (McKenna & Bell, 2007) to identify those who were unsafe to drive, but it was suggested that for elderly people over 70 years a cut-off of > 6 should be used. Using the cut-off > 10 to indicate an unsafe driver, the RDB had a high positive predictive value, in that of those who failed the RDB, 85% were found to be unsafe to drive on the road. However, the ability to detect unsafe drivers was 54%, meaning that of those who were unsafe to drive, only 54% were correctly identified by the RDB (sensitivity 54%, specificity 66%). Using the cut-off recommended for older drivers, with > 6 fails indicating an unsafe driver, the positive predictive value was 78%, but the ability to detect unsafe drivers (sensitivity) was 66% and safe drivers (specificity) 73%. This suggests that for elderly people with dementia the cut-off value of > 6 may be better for detecting unsafe drivers. Therefore for this study two cut-off scores were selected: a cut-off score of > 6 as recommended for individuals over 70 years and a cut-off score of > 10 which is the standard cut-off used to identify unsafe drivers. The advantage of the RDB is that it is available to purchase as a test battery and the administration and scoring procedures are straightforward. However, in the validation sample there were only 53 of 543 (10%) participants with dementia, and the overall decision about safety to drive was informed by the results of the cognitive test. In addition, the participants were all referred for assessment at a Forum-accredited driving assessment centre; they may therefore not be entirely representative of those with dementia who are assessed in clinical practice.

In contrast, the Dementia Drivers' Screening Assessment (DDSA; Lincoln & Radford, 2012) was developed on two samples of people with dementia who were attending memory clinics, the setting where most people are likely to be assessed. The test was validated by comparing performance on a battery of cognitive tests with the Nottingham Neurological Driving Assessment (Lincoln, Taylor, & Radford, 2012), an on-road assessment, blind to the cognitive test results. Discriminant function analysis was used to generate equations to classify patients as safe or unsafe to drive. The equations were found to have good predictive validity for identifying drivers with dementia who were safe to continue driving (Lincoln, Radford, Lee, & Reay, 2006) and this was supported in an independent validation (Lincoln, Taylor, Vella, Bouman, & Radford, 2010). As the decision about safety to drive was made blind to the results of the cognitive assessments, it was not biased to a correspondence. The positive predictive value of the DDSA for detecting unsafe drivers was 82% in the original sample. In the validation sample the positive predictive value for unsafe drivers was lower (62%) The ability to detect safe drivers was better than the ability to detect unsafe drivers. On this basis the test is recommended for deciding who is safe to drive, and those who fail the test should be referred for assessment on the road. In order to administer the DDSA, the materials have to be collated from a range of cognitive tests used in clinical practice, and the scoring procedure is not as straightforward as the RDB. A shortened version of the DDSA, the Nottingham Assessment for Drivers with Dementia (NADD; Lincoln & Radford, 2012) has been developed, which does not require tests from multiple sources, but this also has the disadvantage that the calculation of the recommendation is complex and the NADD is also better at detecting safe drivers than unsafe drivers. The screening properties of these assessments are summarised in Table 1. The RDB and DDSA are comparable in the time taken to administer the test, availability of the equipment and training needed to administer and interpret the assessment.

**TABLE 1:** Screening Properties of Cognitive test batteries for predicting safety to drive.

	Cut-off to	Ability to detect unsafe drivers				
Assessment	detect unsafe drivers	Sensitivity	Specificity	Positive predictive value	Negative predictive value	Overall accuracy
Rookwood Driving Battery	>6	66	73	78	59	57
Rookwood Driving Battery	>10	54	66	85	70	69
Dementia Drivers’ Screening Assessment 2006 sample	<0	90	93	82	96	92
Dementia Drivers’ Screening Assessment 2009 sample	<0	44	89	62	80	76
Nottingham Assessment for Drivers with Dementia Combined 2006 and 2009 samples	<0	54	92	64	89	85

The aim was to compare these two batteries in drivers with dementia.

## METHODS

Ethical approval was granted by the Leicestershire, Northamptonshire and Rutland Research Ethics Committee 1. Research and Development approval was granted by Nottinghamshire Healthcare NHS Trust.

### Participants

Potential participants with dementia were identified through the Mental Health Services for Older People in Nottinghamshire. Clinicians were asked to explain the study to those who were currently driving and to request permission to pass on their contact details to the researchers.

Participants were included in the study if they: (1) were diagnosed with dementia as determined by their treating clinician, (2) had no other medical diagnosis (e.g., stroke, multiple sclerosis) that could affect their performance on the cognitive batteries, (3) had driven a vehicle within the last two years, (4) lived within a 25 mile radius from the recruitment centre, (5) had the capacity to consent to the study as determined by their treating clinician, and (6) consented to take part.

They were excluded from the study if they: (1) did not speak English, as the assessments were standardised in English, (2) were not able to read 12 point text with glasses, as this was used for the information sheet and indicates sufficient vision to see the test materials, and (3) were deaf, as they would not be able to hear the test instructions.

Fifty nine drivers with dementia were identified. Of these, 29 met the criteria and were recruited. Of the 30 that did not meet the criteria: one did not have a diagnosis of dementia, two had other medical conditions and 27 did not consent to take part. Five people withdrew their consent prior to the first assessment and 24 completed the cognitive assessments.

The mean age of participants was 73.0 years (*SD* 8.9, range 51–85) and 18 (75%) were men. Eighteen participants had a diagnosis of Alzheimer's disease, four vascular dementia and two had a dementia of unknown subtype. They had received an average of 12.3 years of education (*SD* 2.9, range 9–18). Participants reported that they had been driving for a mean of 51.5 years (*SD* 10.6). Eleven participants classified themselves as frequent drivers, seven as average frequency drivers and six as infrequent drivers. Two (8%) reported they had had an accident in the last five years.

### Measures

The Rookwood Driving Battery (McKenna, 2009) comprises 12 tests of visual perception, executive and praxis skills:

- Visual Object and Space Perception (VOSP; Warrington & James, 1991) Incomplete Letters, Position Discrimination and Cube Analysis subtests, to assess shape perception and visuospatial abilities.
- Letter Cancellation, in which participants are required to cancel Es and Fs in an array of letters.
- Weigl Sorting Task (Goldstein & Scheerer, 1941) as a measure of abstract thinking.
- Behavioural Assessment of the Dysexecutive Syndrome (BADS; Wilson, Alderman, & Burgess, Emslie, & Evans, 1996): Key Search, Action Programme and the Rule Shift Cards subtests to assess executive function.
- Copying hand movements, Gestures and Use of Objects subtests, involving the miming the use of an object, and copying gestures and hand-movements to assess praxis.
- Tapping and Sequencing to assess rule-bound praxis skills.
- Modified Token Test (Coughlan & Warrington, 1978), a shortened version with eight instructions, as a measure of the ability to follow instructions.
- Letter cancellation (Es and Fs) with a distractor task (threes) as a measure of divided attention.

Each test was scored as pass or fail and a profile score was calculated which provided an overall recommendation about fitness to drive.

The Dementia Drivers' Screening Assessment (Lincoln et al., 2010) comprises the following tests:

- Mini Mental State Examination (Folstein, Folstein, McHugh, & Fanjiang, 2000): total score.
- Stroke Drivers' Screening Assessment (SDSA; Nouri & Lincoln, 1994): Dot Cancellation shortened version (12 lines) time, errors and false positives; Square Matrices Directions and Road Sign Recognition.
- Salford Objective Recognition Test (SORT; Burgess, Dean, Lincoln, & Pearce, 1996) immediate and delayed recognition of words.
- Stroop Color and Word Test (Victoria version: Strauss, Sherman, & Spreen, 2006) scores as the discrepancy between colour–words time and non-colour–words time.
- Visual Object and Space Perception (VOSP; Warrington and James, 1991): Incomplete Letters.
- Behavioural Assessment of the Dysexecutive Syndrome (BADS; Wilson et al., 1996): Rule Shift and Key Search sub-test profile scores.
- Adult Memory and Information Processing Battery (AMIPB; Coughlan & Hollows, 1985): Information Processing A adjusted score.

The NADD comprises the subtests from the SDSA, SORT, Stroop and AMIPB. Apart from the SDSA, all materials are included in the online manual.

The three subtests (BADS Rule Shift, BADS Key Search and VOSP Incomplete Letters) that were in common to both batteries were included in the first battery that was completed.

### Procedure

The RDB and the DDSA were administered over one or two sessions, in an order determined by random allocation. Participants were assessed in their own homes. Demographic and driving details were recorded. This included the number of years driving and the self-reported frequency of driving.

The tests were scored. Those participants who had discrepant recommendations about safety to drive were invited to be assessed on the road. They were excluded from this stage of the study if they did not have had a valid driving licence. Those who agreed were assessed on the road by an approved driving instructor experienced in assessing people with dementia, who was blind to the cognitive test results. The approved driving instructor met the participants and provided them with an overview of the driving assessment and answered any questions. The on-road assessments were conducted using the participants' own cars. They were assessed on the Nottingham Neurological Driving Assessment. This is a standardised on-road assessment which comprises 25 road manoeuvres, such as turning left and merging with traffic on main roads. It was conducted on a pre-planned route which included quiet roads, dual carriageways and busy town roads. Each manoeuvre was recorded as correct, minor error (no effect on safety) and major error (compromising safety). At the end of the drive participants were graded as “definitely unsafe”, “probably unsafe”, “probably safe” or “definitely safe” to drive. The driving assessment lasted approximately 40 minutes.

Although the NNDA assessment was planned for all participants with discrepant results, one participant chose to arrange his own driving test with an independent approved driving instructor who completed the NNDA retrospectively.

## RESULTS

Mann-Whitney *U*-tests were conducted to assess whether administration of either the RDB (Group 1) or the DDSA (Group 2) first had a significant effect on participants' performance. No significant differences in performance were found between the two groups on any measure (*p* = .06–1.0).

The distribution of participants' scores on the RDB and DDSA are shown in Table 2. The overall conclusions from each battery were cross-tabulated and the results are shown in Table 3.

**TABLE 2:** Distribution of scores on cognitive assessments

Test	Subtest	Possible range	Median	Inter-quartile range	Number Passed	
					*n* %	
VOSP	Incomplete letters	0–20	19	17–20	15	63
	Position discrimination	0–20	20	19–20	20	83
	Cube analysis	0–10	9	6–10	16	67
Letter cancellation	Target number	0–86	41.5	29–70	14	58
	Percentage errors	0–100	2.3	0–61	22	92
Weigl Sorting		0–4	4	2–4	14	58
BADS	Key search	0–16	9	5–14	7	71
	Action programme	0–5	4	4–5	19	79
	Rule shift	0–20	15.5	10–20	12	50
Copying gestures and objects	No. correct	0–16	16	15–16	22	92
Tapping and sequencing	No. correct	0–15	13.5	10–15	16	67
Letter cancellation with distractor	Target number reached	0–86	21.5	4–53	10	42
	Percentage error	0–100	1.6	0–9	20	83
	Threes	0–10	2.5	0–8	10	42
Modified Token Test		0–8	6	5–7	11	46
Overall Rookwood Driving Battery		0–22	8	4–11		
						
MMSE	Score	0–30	26	23–27		
SDSA Dot cancellation	Time for 12 lines	0–450	258	233–432		
	Errors in 12 lines	0–98	10.5	5–17		
	False positives in 12 lines	0–202	0	0–0		
SDSA Directions		0–32	15	6–20		
SDSA Road sign recognition		0–12	5	2–8		
SORT Words	Immediate	0–12	10	7–12		
	Delayed	0–12	7	5–11		
Stroop	Interference score	0–300	33	10–44		
AMIPB Information Processing	Adjusted score	0–630	38.3	25–60		
Overall discrepancy		–100–+100	23.3	17–35		

VOSP = Visual Object and Space Perception Battery, BADS = Behavioural Assessment of the Dysexecutive Syndrome, SDSA = Stroke Drivers’ Screening Assessment, MMSE = Mini-Mental State Examination, SORT = Salford Objective Recognition Test, AMIPB = Adult Memory and Information Processing Battery.

**TABLE 3:** Classification of drivers on the basis of cognitive tests

		Rookwood Driving Battery		
		Fail	Pass	Agreement
			Score > 10	Score 0–10	
Dementia Drivers’ Screening Assessment	Fail	3	1	Kappa = .36	
	Pass	5	15	*p* = .05	
				75% agreement	
		Fail	Pass		
		Score > 6	Score 0–6		
Dementia Drivers’ Screening Assessment	Fail	4	0	Kappa = .21	
	Pass	11	9	*p* = .09	
				54% agreement	
		Fail	Pass		
		Score > 10	Score 0–10		
Nottingham Assessment for Drivers with Dementia	Fail	3	0	Kappa = .44	
	Pass	5	16	*p* = .009	
				83% agreement	
		Fail	Pass		
		Score > 6	Score 0–6		
Nottingham Assessment for Drivers with Dementia	Fail	3	0	Kappa = .16	
	Pass	12	9	*p* = .15	
				50% agreement	

Using the recommended cut-off of > 6 on the RDB for elderly people, 9 (38%) participants were classified as a pass on both tests, 4 (17%) participants were classified as a fail on both tests, and 11 (46%) were classified as a pass by the DDSA and fail by the RDB. No participants were classified as a pass by the RDB and fail by the DDSA. There was agreement on 13 out of 24 participants (54%). The level of concordance between the tests was Kappa = .21 (*p* = .09), a poor level of agreement (Fleiss, Levin, & Paik, 2003).

The classifications of the RDB and DDSA, using a cut-off score of > 10 on the RDB, are also shown in Table 3. Fifteen (63%) participants were classified as a pass on both tests and three (13%) participants were classified as a fail on both tests. There were five (21%) that failed the RDB and passed the DDSA and one (4%) that passed the RDB and failed the DDSA. There was agreement on 18 out of 24 participants (67%). The level of concordance between the tests was Kappa = .36 (*p* = .05), a poor level of agreement (Fleiss et al., 2003).

The relation between the NADD and the RDB is also shown in Table 3. There was 85% agreement between the classification of the NADD and the RDB using a cut-off score of > 10. This was a moderate level of agreement (Kappa .51, *p* = .005). Using the cut-off > 6 on the RDB showed lower agreement with the NADD, 52%, which is also a poor level of agreement. (Kappa = .18, *p* = .14).

Using a cut-off > 10 on the RDB, there were six participants whose RDB and DDSA results did not agree. Of these, five were predicted to pass on the basis of the DDSA but fail on the basis of the RDB. Three of these completed the NNDA. One participant was predicted to fail on the basis of the DDSA, and to pass on the basis of the RDB. This person did not consent to be assessed on the road.

The participants who were assessed on the road were all men, aged 74, 77, and 78 years, respectively. Two had Alzheimer's dementia and one had vascular dementia. They had been driving for 54, 61, and 45 years, respectively. They had had no history of accidents or traffic violations in the preceding five years. They were all predicted to be unsafe on the road using the RDB cut-off score of > 10; their scores were 11, 14, and 15, respectively. The predicted difference score on both the DDSA (2.86, 5.03, and 5.69) and NADD (1.46, 1.49, and 2.37) was positive, indicating they were predicted to be safe to drive. All three were assessed as safe to drive on the road test.

## DISCUSSION

These results highlight discrepancies in the classifications of the RDB and DDSA. The findings suggest that the RDB is classifying individuals as unsafe to drive, who are classified as safe by the DDSA. When viewed in the context of previous research, this is unexpected as both batteries have been validated as predicting on-road driving abilities. However, the differences in classifications may in part be explained in terms of their validation samples. The RDB was validated on people with dementia who were referred to a specialist driving assessment centre and thus were likely to be individuals at the more severe end of the spectrum whose driving abilities were questioned (McKenna & Bell, 2007). This is supported by Radford (2000), who also recruited participants with dementia from a driving assessment centre, and found a high rate of failure on neuropsychological assessments, and most participants with dementia were found to be unsafe to drive. According to Radford (2000), only those participants whose safety to drive is questioned or who have lost insight and have not been persuaded to stop driving are referred for specialist on-road assessment.

The RDB was developed for use in assessment centres with a main focus on identifying individuals who are unsafe to drive (McKenna & Bell, 2007). It has a high positive predictive value, so that those who fail the test are likely to be unsafe drivers. The low sensitivity to fails was not a problem in this context as all participants were also assessed on the road, so the test was used mainly to highlight cognitive problems that may impact on driving ability, rather than to make decisions about the ability to drive. In contrast, the DDSA was developed for use within a clinic setting where the primary aim was to identify those who were safe to continue driving (Lincoln et al., 2006). Most individuals were attending a memory clinic for diagnosis and thus were likely to have less severe cognitive problems. This was also an explanation offered for the high proportion of safe drivers in the validation sample (Lincoln et al., 2010). The test is used in this context to identify those who are safe to continue driving, any others are recommended to consider retiring from driving or to be assessed on the road.

The correspondence between the two batteries was highest when using the original cut-off score of > 10 on the RDB to indicate an unsafe driver. The present results do not support the use of the > 6 cut-off for people with dementia, even though many of these were over 70 years. The findings also support the use of the NADD as a short version of the DDSA, as the correspondence with the RDB was greater for the NADD than the DDSA.

Three drivers failed both the RDB and the DDSA. Five drivers failed the RDB and passed the DDSA and of these, three were assessed on the road and found to be safe to drive. This suggests that the RDB is stricter than the DDSA and may be leading to some people with dementia being stopped from driving prematurely. One of the participants who was not assessed on the road had a very atypical pattern of results and would probably have been considered unsafe to drive on the basis of individual test scores. His RDB score was 19 indicating that he was impaired on most tests of the RDB. In addition, he performed poorly on all tests of the DDSA. The overall pass and fail equations were both negative, which is unusual. It suggests that his scores may have been so far outside the range of scores of participants in the original validation study that the equations did not perform as expected. This anomaly has also been noted clinically in stroke patients with severe visual neglect who pass the Stroke Drivers' Screening Assessment. The explanation offered is that those with neglect were excluded from the validation studies and therefore the equations are not appropriate for use with patients with severe neglect. One person passed the RDB but failed the DDSA. Inspection of this participant's scores revealed that he was in the borderline category on the RDB, score 8, and failed the DDSA, mainly because of very low scores on the Road Sign Recognition and Stroop tests. These are both measures of executive abilities and it may be that the RDB did not detect these executive problems.

There are limitations to the study. It would have been desirable for all participants to be assessed on the road independently of their performance on the two cognitive screening batteries. However, resources were not available to do this. Also of those with discrepant results, only half agreed to be assessed on the road. This related to concerns about losing their driving licence, since, if found to be definitely unsafe to drive, there was a duty of care to notify the psychiatrist or general practitioner responsible for their care if the participants chose not to. Participants were recruited by referral from psychiatrists. The criteria included that they should have a diagnosis of dementia, but there was no information on how the diagnosis was made. They were also required to have driven within the previous two years, which meant that some may not have driven for a long time and some may have given up driving relatively recently. It was considered that even if they had given up driving the recommendations from the two cognitive tests should be consistent with each other and all those assessed on the road were required to have a valid driving licence.

Overall, these findings highlight the importance of examining the screening properties of assessments before using them to make clinical decisions. It seems that those who fail the RDB are likely to be unsafe on the road. The cut-off score of more than 10 provides recommendations which are more consistent with the DDSA. However, not all unsafe drivers will be detected by the RDB. The DDSA is more likely to classify drivers as safe and is better at identifying safe drivers than unsafe drivers. It is therefore suggested than anyone failing the DDSA should be assessed on the road.
